# Comparative Degumming of Rapeseed Oil: Phospholipid Recovery, Bioactive Lipid Concentrations, and Molecular Remodeling

**DOI:** 10.3390/molecules31173099

**Published:** 2026-09-04

**Authors:** Hongli Huang, Fangcheng Shao, Xinlei Feng, Jun Jin, Siyu Zhang

**Affiliations:** 1 College of Food Science and Engineering, Northwest A&F University, Yangling 712100, China; 2 Shandong Animal Products Quality and Safety Center, Jinan 250100, China; 3 State Key Laboratory of Food Science and Resources, School of Food Science and Technology, Jiangnan University, Wuxi 214122, China

**Keywords:** rapeseed oil, phospholipids, ethanol degumming, metabolomics, lipidomics

## Abstract

Rapeseed oil is an important edible oil with a favorable fatty acid composition and contains endogenous phospholipids (PLs) and lipid-soluble minor components that are relevant to refining, oxidative stability, and product quality. In this study, water degumming (WDG), citric acid degumming (ADG), and ethanol degumming (EDG) were systematically evaluated for their effects on oil quality, phospholipid recovery, and bioactive lipid concentrations. A modified Folch extraction was included solely as a laboratory solvent-extraction benchmark for the compositional characterization of phospholipid-rich fractions. WDG and ADG showed the highest phosphorus removal efficiency, reducing phosphorus to undetectable levels, whereas EDG also achieved substantial dephosphorization. ADG produced the highest PL yield (83.22%), while WDG gave the highest oil recovery (98.42%) and PL purity (95.49%). Although the bulk FA composition of the oil remained largely unchanged after treatment, the recovered PL fractions showed clear differences in FA distribution and PL subclass composition. EDG yielded the highest phosphatidylcholine (PC) and lysophosphatidylcholine (LPC) contents, indicating selective enrichment of choline-containing PLs. The concentrations of carotenoids, tocopherols, and phytosterols were also process-dependent. Untargeted metabolomic and lipidomic analyses further showed that EDG induced distinct compositional remodeling relative to crude rapeseed oil. Overall, WDG was preferable for oil retention, ADG for maximum PL recovery, and EDG provided a distinct balance between oil recovery, PL recovery, and enrichment of PC- and LPC-containing fractions.

## 1. Introduction

Rapeseed oil (RO) is one of the most important edible vegetable oils, due to its favorable fatty acid (FA) composition, particularly its high proportion of unsaturated fatty acids (FAs). In addition to triacylglycerols (TAGs), crude RO also contains a wide range of endogenous minor components, including phospholipids (PLs), tocopherols, phytosterols, carotenoids, monoacylglycerols (MAGs), diacylglycerols (DAGs), free fatty acids (FFAs), and pigments [1,2,3,4]. These components are closely related to the nutritional value, oxidative stability, color, and processing behavior of the oil. Some are desirable, as they contribute to antioxidant activity and nutritional functionality, whereas others, especially polar compounds and gum-forming substances, such as PLs, complicate refining and reduce the storage stability of crude oil [2,5,6]. As a consequence, refining is required before RO can be used in most food applications.

The first key step in RO refining is degumming, which is mainly intended to remove PLs and other gum-forming compounds from crude oil [4,5,6,7]. In practice, however, degumming is more than a simple purification step. It also affects the distribution of amphiphilic lipids and other minor compounds between the oil phase and the recovered gum fraction. Water degumming (WDG) is generally regarded as a mild process that mainly removes hydratable PLs, whereas acid-assisted degumming (ADG) improves the removal of non-hydratable PLs by disrupting metal–PL associations and converting part of them into more readily separable forms [4,5,6,7,8]. Ethanol-assisted degumming (EDG) may provide a different separation environment, since ethanol changes phase polarity and may alter the partitioning behavior of PLs and related micronutrients.

Beyond conventional WDG and ADG, recent technological developments have increasingly focused on process intensification and selective enzymatic conversion of PLs [4,7,8]. Hydrodynamic cavitation, sometimes referred to as nanodegumming in industrial contexts, enhances oil–water interfacial disruption, turbulence, and mass transfer, thereby accelerating PL hydration and phase separation [7]. Enzymatic degumming represents another important technological direction [4,8]. Phospholipases A_1_ and A_2_ convert phospholipids into more hydratable lysophospholipids, whereas phospholipase C cleaves the phosphodiester bond and retains diacylglycerol in the oil phase. Modern strategies also combine phospholipases with ultrasound, high-shear mixing, membrane separation, or other intensified operations to improve phosphorus removal, oil recovery, and process sustainability. These developments provide the broader technological context for edible-oil degumming, although the present study focuses specifically on the composition and recovery of PL-rich fractions obtained through water-, acid-, and ethanol-assisted treatments.

This issue is particularly relevant in RO because PLs should not be regarded only as undesirable refining impurities. They are structurally and nutritionally important amphiphilic lipids. Among them, choline-containing PLs, especially phosphatidylcholine (PC), are of particular interest as choline is required for normal physiological function and has been discussed in relation to neurological development and brain function [9,10,11,12]. More broadly, dietary PLs have been associated with membrane function and cognitive processes across the lifespan [9,10,13].

Besides PLs, the retention of other bioactive lipid components during degumming is also important. Tocopherols are major endogenous antioxidants in vegetable oils, carotenoids contribute to both color and antioxidant properties, and phytosterols are valuable unsaponifiable constituents [1,2,3,4,14]. Previous studies on RO refining have shown that processing can significantly affect tocopherols, carotenoids, and other micronutrients, even when the bulk FA composition remains largely unchanged [2]. At the same time, oxidative stability is controlled by multiple factors, including FA unsaturation, hydroperoxide decomposition, trace metals, endogenous antioxidants, pigments, and processing history [15,16,17]. Thus, evaluating degumming only by phosphorus removal or oil recovery is too limited. A more informative assessment should include PL recovery, PL class distribution, retention of phospholipid fractions and bioactive minor lipids, and the resulting changes in oil quality.

Previous studies have optimized water and enzymatic degumming of RO and evaluated their effects on residual phosphorus, oil yield, oxidative stability, bioactive component retention, acylglycerol composition, and PLs remaining in the degummed oil [4,18]. However, less attention has been given to the recovered PL-rich fractions as potential value-added products, particularly their recovery yield, purity, fatty acid distribution, subclass composition, physical morphology, and molecular-species profile. Moreover, the selective effects of ethanol-assisted degumming on PL recovery and broader lipid remodeling remain insufficiently characterized.

Accordingly, WDG, ADG, and EDG were systematically evaluated for their effects on oil quality, PL recovery, and bioactive lipid retention. A Folch extraction [19] was included solely as a laboratory solvent-extraction benchmark for the compositional characterization of PL-rich fractions. Crude RO and the corresponding treated oils or recovered fractions were systematically evaluated in terms of FA composition, MAGs, DAGs, phosphorus content, peroxide value (PV), oxidation induction time (OIT), color parameters, carotenoids, tocopherols, phytosterols, PL yield, oil recovery, PL purity, and PL class distribution. The objective of this study was to clarify how different degumming strategies influence PL recovery and bioactive lipid retention in RO, and to identify processes that better balance oil refining with the value-added recovery of nutritionally relevant lipid fractions. The novelty of this work lies in the integrated evaluation of both oil quality and the recovered PL-rich fractions, including PL yield and purity, oil recovery, PL subclass and FA distribution, microstructure, and retention of lipid-soluble bioactive components. Untargeted metabolomic and lipidomic analyses were further performed to characterize the compositional remodeling associated with ethanol degumming at the metabolite and molecular-species levels.

## 2. Results and Discussion

### 2.1. Effects of Degumming on Phosphorus Removal and Oil Quality

As shown in Table 1, crude rapeseed oil contained 0.249 ± 0.017 mg phosphorus per gram of oil, indicating a moderate initial PL load. All treatments reduced phosphorus markedly, although the extent of removal depended on the process. WDG and ADG reduced phosphorus to undetectable levels, EDG lowered it to 0.022 ± 0.001 mg/g, and Folch extraction reduced it to 0.185 ± 0.004 mg/g. The incomplete phosphorus removal by EDG likely reflects its different separation mechanism. Unlike WDG, which promotes hydration of hydratable PLs, and ADG, which facilitates removal of non-hydratable PLs through disruption of metal–PL associations, EDG depends primarily on solvent-polarity-driven partitioning and aggregation. Under the ethanol concentration and phase-separation conditions used in this study, a small proportion of phosphorus-containing compounds apparently remained in the oil-rich phase. These results confirm that all three degumming treatments were effective, with WDG and ADG showing the highest dephosphorization efficiency. The lower residual phosphorus after WDG and ADG agrees with their established ability to remove hydratable and non-hydratable PLs, respectively [4,5,6,7,8,18,20]. However, phosphorus removal alone did not predict oil recovery, oxidative stability, or the composition of the recovered PL fraction, indicating that the processes should be evaluated using a broader set of recovery and quality indices.

The oxidation-related indices further showed that degumming changed oil quality beyond phosphorus removal. According to Table 1, crude rapeseed oil had the lowest peroxide value (PV, 0.37 mEq/kg) and the highest oxidation induction time (OIT, 181.13 min), indicating the greatest oxidative resistance among all samples. After treatment, PV increased and OIT decreased in all cases. Among the degummed oils, ADG and WDG showed relatively low PVs (1.74 and 1.79 mEq/kg, respectively), whereas EDG gave the highest PV (4.02 mEq/kg). However, EDG also showed the highest OIT among the degummed oils (108.90 min), higher than WDG (101.37 min), ADG (100.53 min), and Folch (57.60 min). This indicates that PV and OIT should not be interpreted independently. Similar differences between primary oxidation indices and induction-based stability measurements have been reported for rapeseed oil and canola oil during processing and storage [16,21,22]. These findings are not inherently contradictory because PV quantifies hydroperoxides already present at the time of measurement, whereas OIT reflects resistance to subsequent oxidation under accelerated conditions [15,16,17]. The elevated PV of EDG may indicate limited primary oxidation during high-shear mixing, solvent contact, and phase separation. Its slightly longer OIT relative to WDG and ADG may reflect a different balance among residual antioxidants, PLs, trace pro-oxidants, and oxidation products. However, trace metals and secondary oxidation products were not measured. Therefore, metal removal or altered hydroperoxide decomposition should be regarded as possible explanations rather than demonstrated mechanisms. Importantly, all PVs remained below 5 mEq O_2_/kg, indicating a low absolute degree of primary oxidation. The observed differences are therefore useful for comparing immediate process effects but do not independently establish meaningful differences in shelf life. Controlled storage experiments would be required to determine practical oxidative stability.

Taken together, WDG and ADG were the most effective in terms of phosphorus removal. EDG retained a slightly longer OIT than WDG and ADG despite its higher initial PV, indicating that phosphorus removal and oxidation-related indices represent distinct process outcomes.

### 2.2. Oil Recovery and PL Yield and Purity

**Figure 1:**
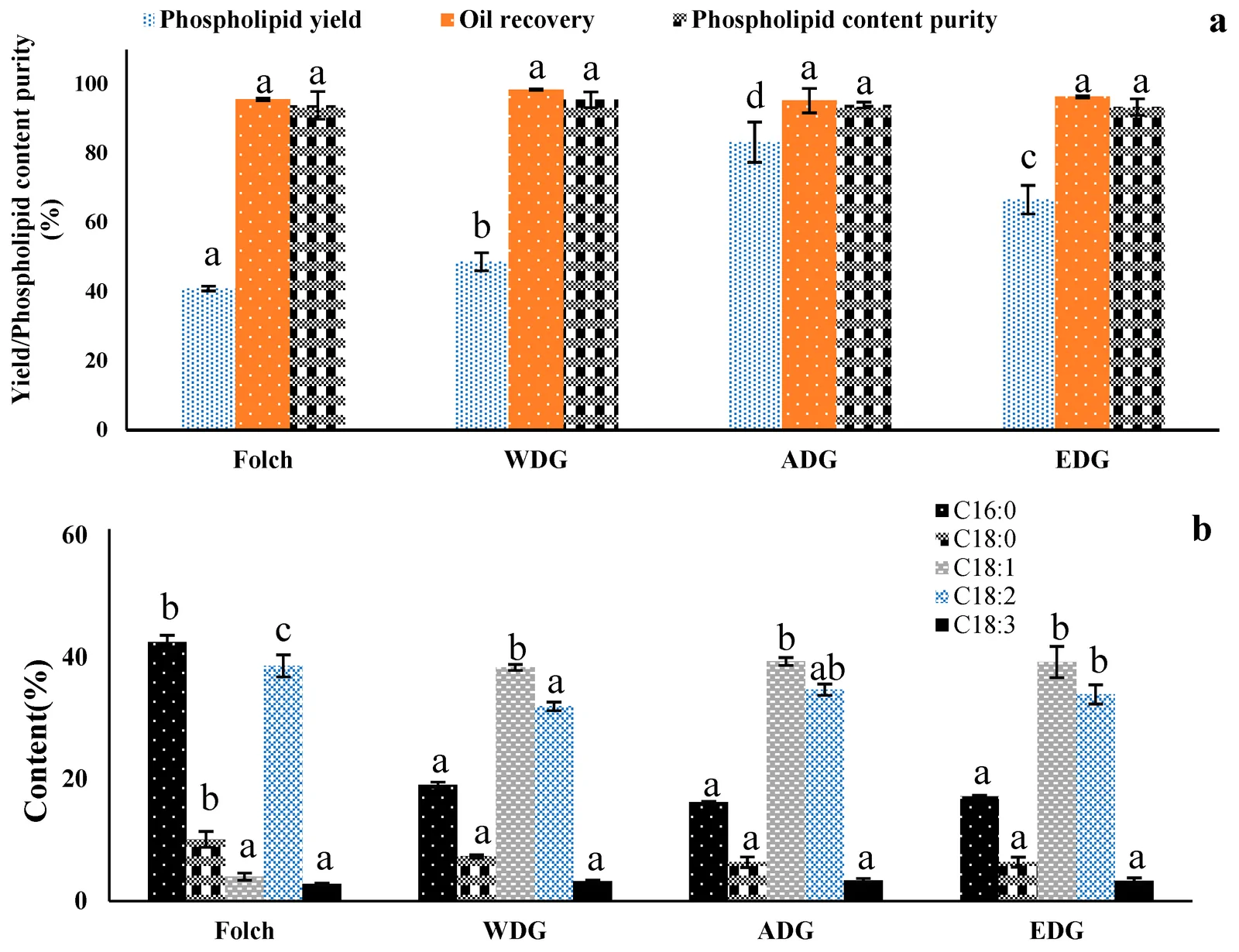
Effects of different degumming processes on phospholipid (PL) recovery and fatty acid composition of recovered phospholipid fractions. (**a**) PL yield, oil recovery, and PL purity obtained by Folch extraction, water degumming (WDG), citric acid degumming (ADG), and ethanol degumming (EDG). (**b**) Fatty acid composition of the recovered PL fractions produced by different methods. Data are expressed as mean ± standard deviation. Different letters indicate significant differences among treatments (*p* < 0.05).

The recovery and quality of the PL-rich fractions are important for the value-added utilization of rapeseed oil gums. As shown in Figure 1a, the four treatments differed clearly in the balance between oil retention and PL recovery. WDG gave the highest oil recovery (98.42%), followed by EDG (96.36%), Folch (95.59%), and ADG (95.24%). In contrast, ADG produced the highest PL yield (83.22%), followed by EDG (66.60%), WDG (48.63%), and Folch (40.90%). Thus, the process that recovered the greatest amount of PL was not the one that best preserved the oil phase. This trade-off is consistent with the general behavior of degumming systems, since acid-assisted treatment usually promotes more extensive PL separation than simple hydration, but stronger PL removal is often accompanied by greater oil entrainment into the gum phase [5,6,7,8]. Similar trends have been observed in soft-degumming and electrolyte-degumming studies, where improved gum removal was accompanied by changes in oil retention and minor-component distribution [4,23,24].

PL purity remained high in all recovered fractions. As shown in Figure 1a, purity ranged from 93.38% to 95.49%, with WDG giving the highest value. This indicates that all methods were able to produce concentrated PL-rich fractions. However, yield, purity, and oil recovery should be considered together rather than separately. ADG is preferable when the main goal is maximum PL recovery, WDG is more suitable when minimizing oil loss is prioritized, and EDG provides a compromise between the two. From a practical standpoint, this intermediate behavior makes EDG attractive when both PL recovery and oil retention are important. Recent studies on rapeseed oil processing have likewise emphasized that separation strategy can determine whether refining byproducts are treated as waste streams or as potential sources of value-added PLs and other minor lipids [4,23].

Considering these 3 factors together, EDG provided an intermediate balance between oil recovery and PL recovery while producing a PL-rich fraction with relatively high PC and LPC proportions at laboratory scale. These findings identify a potentially useful process window rather than a validated continuous industrial process. Translation to industrial operation will require pilot-scale evaluation of continuous mixing and phase separation, ethanol recovery and recycling, solvent safety, energy consumption calculation, and reproducibility evaluation of PL subclass enrichment.

### 2.3. Fatty Acid Composition of Recovered Phospholipid Fractions

The FA composition of the oil phase remained essentially unchanged after Folch extraction and after the three degumming treatments. As summarized in Table 1, oleic acid (C18:1) remained the dominant FA at about 57.5%, followed by linoleic acid (C18:2) at about 24.4%, whereas palmitic acid (C16:0), stearic acid (C18:0), and linolenic acid (C18:3) varied only slightly among samples. This indicates that the major triacylglycerol matrix of rapeseed oil was preserved during treatment. Similar observations have been reported in refining studies of rapeseed oil, in which the bulk FA profile remained relatively stable while the minor-component composition changed much more strongly [1,2,3,4].

By contrast, the FA distribution of the recovered PL fractions differed among methods. As shown in Figure 1b, the PL fractions recovered by WDG, ADG, and EDG were generally less enriched in C16:0 and more enriched in unsaturated acyl chains, especially C18:1 and C18:2, than the Folch fraction. This suggests that the different processes did not recover identical PL populations, but instead selectively enriched PLs with different molecular characteristics. Since PL acyl-chain composition affects polarity, oxidation behavior, and interfacial performance, these differences are likely to influence the functional properties of the recovered PL fractions [4,13]. This interpretation is also consistent with lipidomic analyses of cold-pressed rapeseed oil, which showed that lipid molecular composition can vary substantially even when the overall oil matrix appears similar at the bulk level [25].

### 2.4. Phospholipid Class Distribution, Color Characteristics, and Morphology

**Figure 2:**
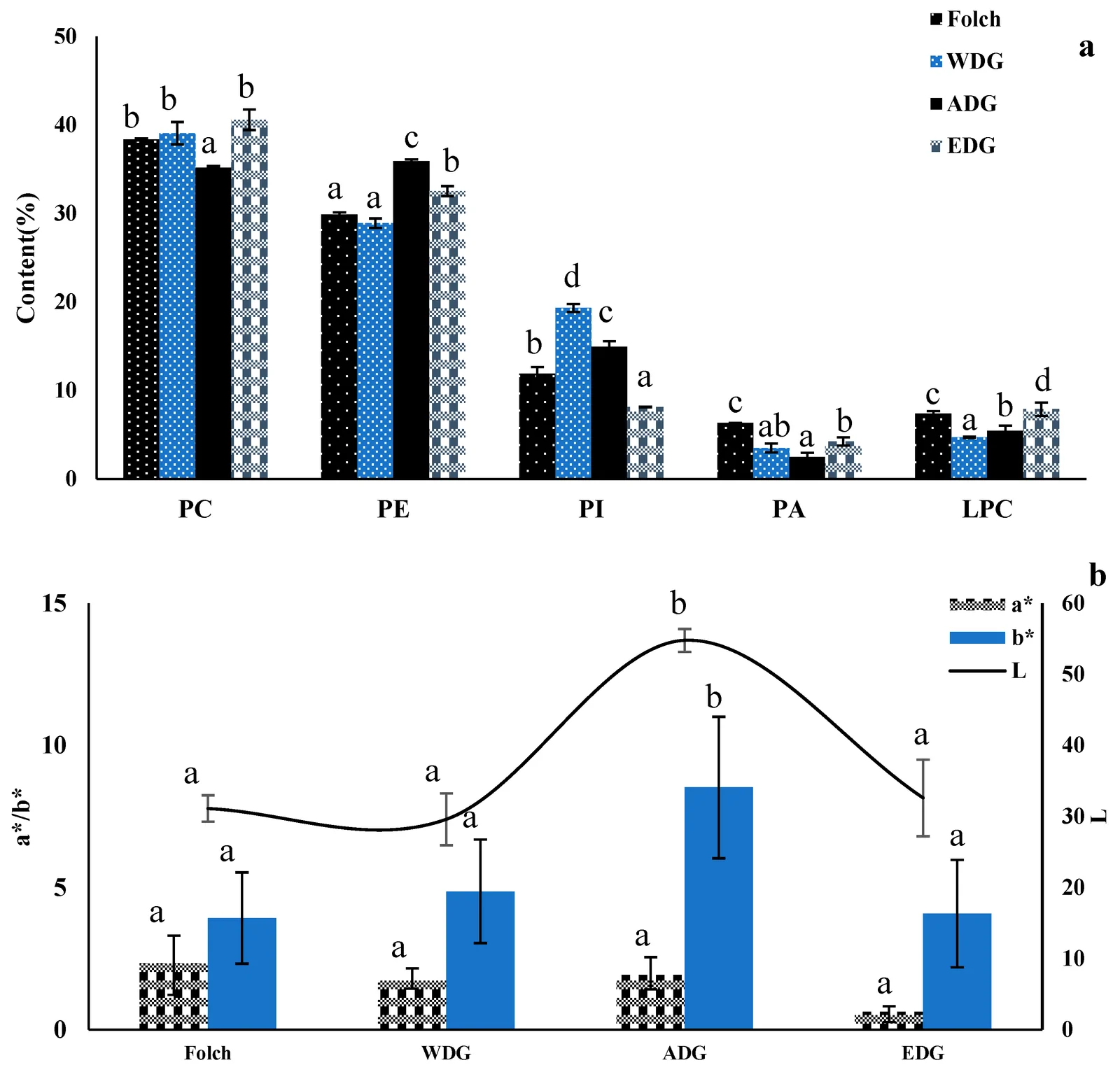
Effects of different degumming processes on the phospholipid class distribution and color characteristics of recovered phospholipid fractions. (**a**) Relative contents of phosphatidylcholine (PC), phosphatidylethanolamine (PE), phosphatidylinositol (PI), phosphatidic acid (PA), and lysophosphatidylcholine (LPC) in phospholipid fractions obtained by Folch extraction, water degumming (WDG), citric acid degumming (ADG), and ethanol degumming (EDG). (**b**) Color parameters of the recovered phospholipid fractions, including L, a*, and b*. Data are expressed as mean ± standard deviation. Different lowercase letters indicate significant differences among treatments for the same parameter (*p* < 0.05).

The PL subclass data further showed that the recovered fractions were compositionally distinct. As shown in Figure 2a, phosphatidylcholine (PC) and phosphatidylethanolamine (PE) were the dominant PL classes in all samples, but their relative abundance varied substantially among methods. Folch gave a profile containing 38.37% PC and 29.88% PE. WDG showed a similar PC content (39.07%) but the highest phosphatidylinositol (PI) content (19.32%). ADG reduced PC to 35.18% and increased PE to 35.92%. EDG produced the highest PC content (40.59%) and the highest lysophosphatidylcholine (LPC) content (7.89%), while PI decreased to 8.13%. This selective profile may reflect the combined effects of the aqueous ethanol solvent environment, differential solubility and interfacial partitioning of PL subclasses, and low-temperature phase separation. Because these factors were not independently varied, the enrichment cannot be attributed to a single EDG parameter. These results indicate that the different treatments were selective not only in total PL recovery but also in PL subclass composition.

This is particularly relevant because choline-containing PLs, especially PC, are nutritionally important and have been discussed in relation to membrane function, choline supply, and cognition-related nutrition [9,10,11,12]. More broadly, dietary PLs and polar lipids have been associated with structural and functional roles in neural development and cognitive maintenance [13,26,27]. From this perspective, EDG is particularly noteworthy because it favored recovery of a PC- and LPC-enriched fraction. ADG, in contrast, was more effective for maximizing total PL yield, whereas WDG provided the purest PL fraction and favored PI enrichment.

The apparent color parameters varied among treatments, but some measurements also showed substantial between-replicate variability. In particular, ADG showed an L* value of 12.41 ± 10.64, while the Folch-treated samples showed an a* value of 2.95 ± 2.04. Re-examination of the original data confirmed that these values were neither transcription errors nor statistically removable outliers. Instead, the variability reflected visible turbidity and incomplete clarification of the corresponding oil fractions.

Suspended particles and residual dispersed phases can produce heterogeneous light scattering and strongly affect instrumental color measurements. Consequently, the lower mean L* value of ADG should not be interpreted solely as greater pigment-related darkening, and the Folch a* value should not be interpreted as a uniform shift toward redness. These measurements describe the apparent appearance of the samples after processing, including the contribution of residual turbidity.

For this reason, differences in apparent color were interpreted cautiously and were not used independently to infer pigment removal or retention. A separate measurement after standardized clarification would be required to distinguish intrinsic oil color from turbidity-related optical effects.

**Figure 3:**
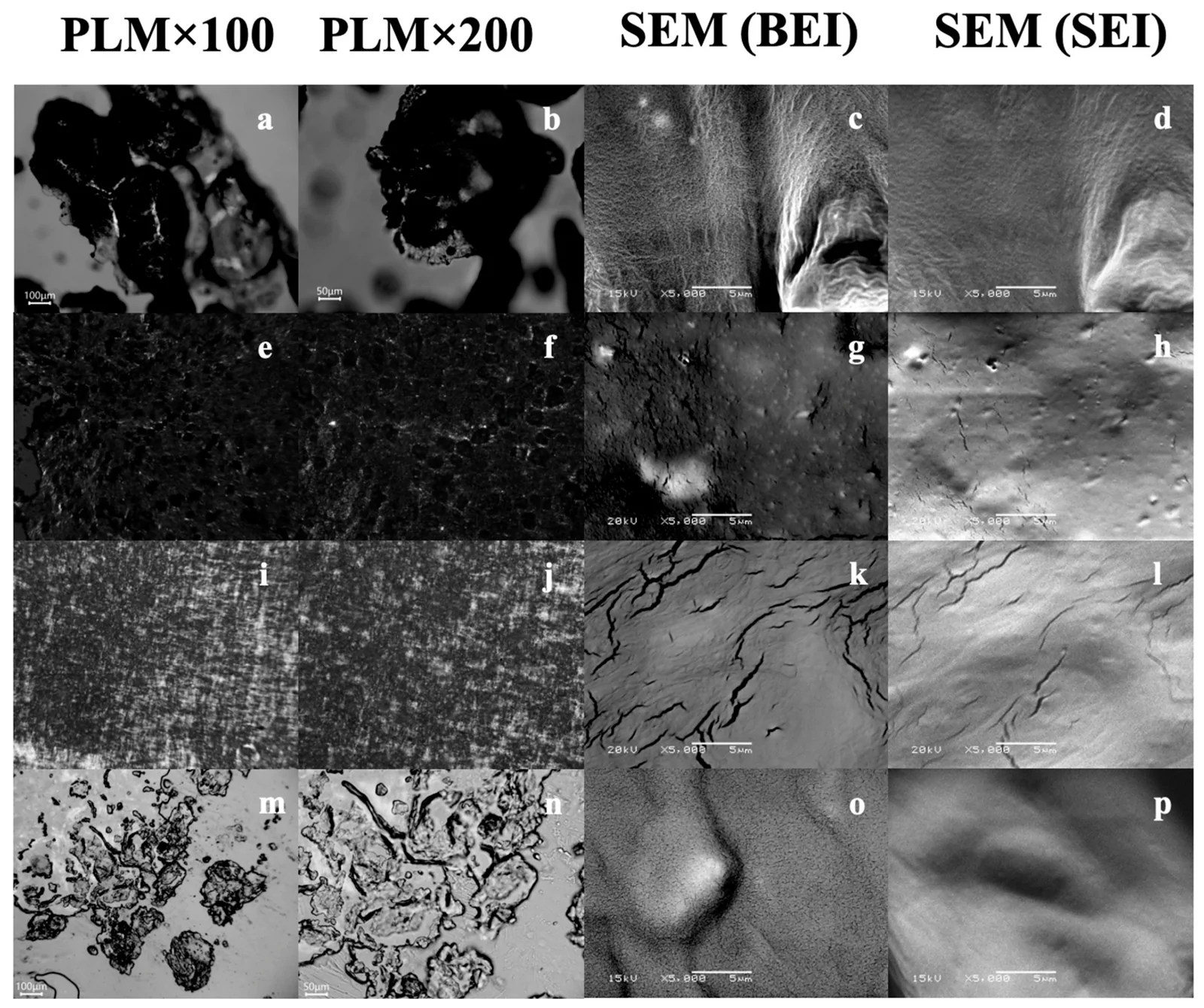
Microstructural morphology of phospholipid fractions recovered by different degumming methods. The first to fourth rows correspond to Folch extraction, water degumming (WDG), citric acid degumming (ADG), and ethanol degumming (EDG), respectively. The first two columns show polarized light microscopy (PLM) images at different magnifications, while the third and fourth columns show scanning electron microscopy (SEM) images obtained in backscattered electron imaging (BEI) and secondary electron imaging (SEI) modes, respectively. Panels (**a**–**d**), (**e**–**h**), (**i**–**l**), and (**m**–**p**) represent Folch, WDG, ADG, and EDG samples, respectively.

As shown in Figure 3, the morphology of the recovered PL was further examined by polarized light microscopy (PLM) and scanning electron microscopy (SEM). The PLM images showed that all recovered fractions formed heterogeneous aggregated structures, but their dispersion and domain morphology differed among treatments. Folch and WDG fractions showed relatively compact and dark aggregates, whereas ADG and EDG displayed more dispersed and irregularly distributed structures. SEM-BEI and SEM-SEI observations further revealed treatment-dependent differences in surface morphology, including compact regions, cracks, layered structures, and uneven surfaces. These microstructural differences suggest that the recovery process affected not only the chemical composition of the phospholipid-rich fractions but also their physical organization, which may influence their subsequent dispersion and application as functional lipid ingredients.

### 2.5. Concentrations of Carotenoids, Tocopherols, and Phytosterols

**Figure 4:**
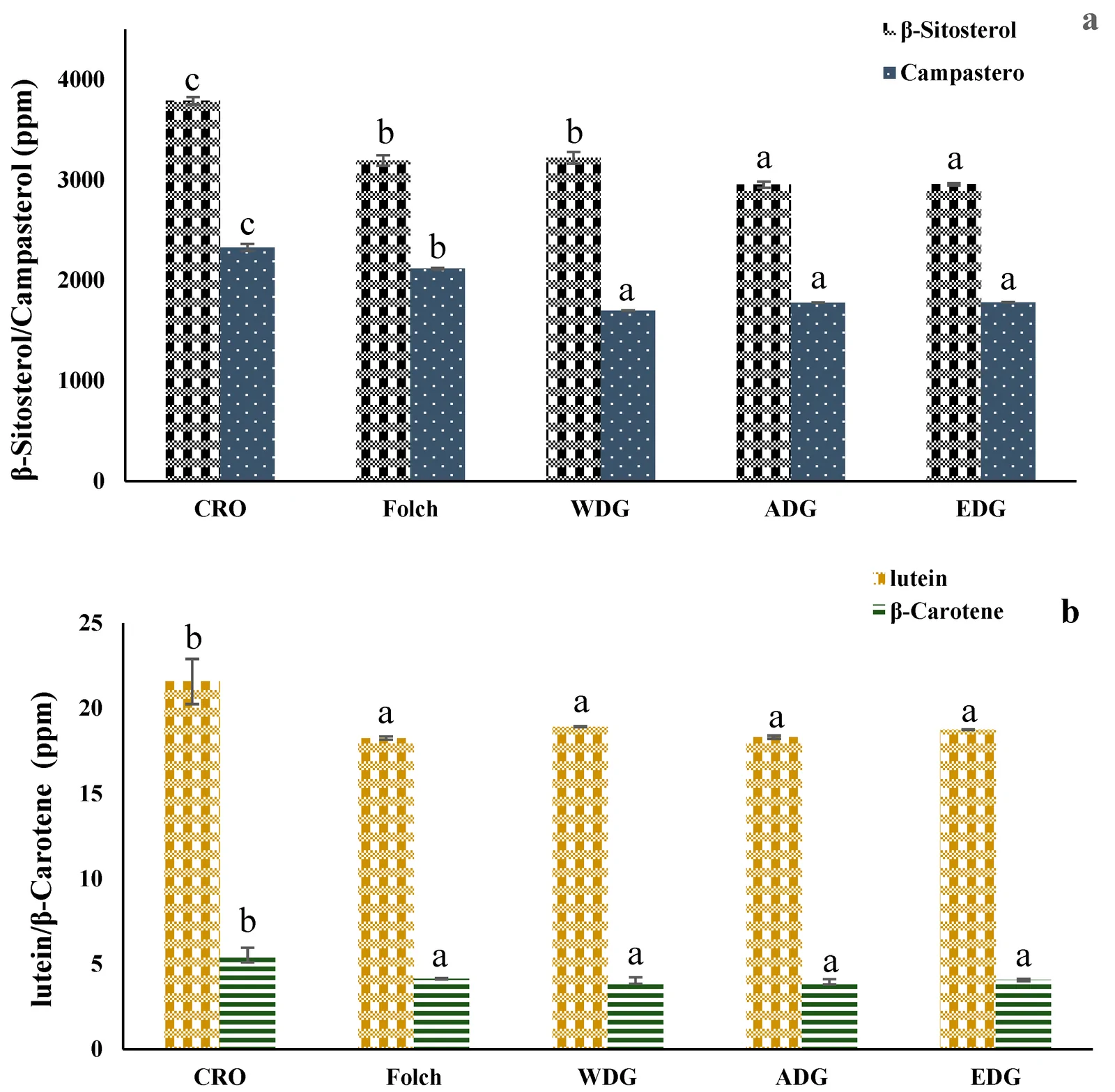

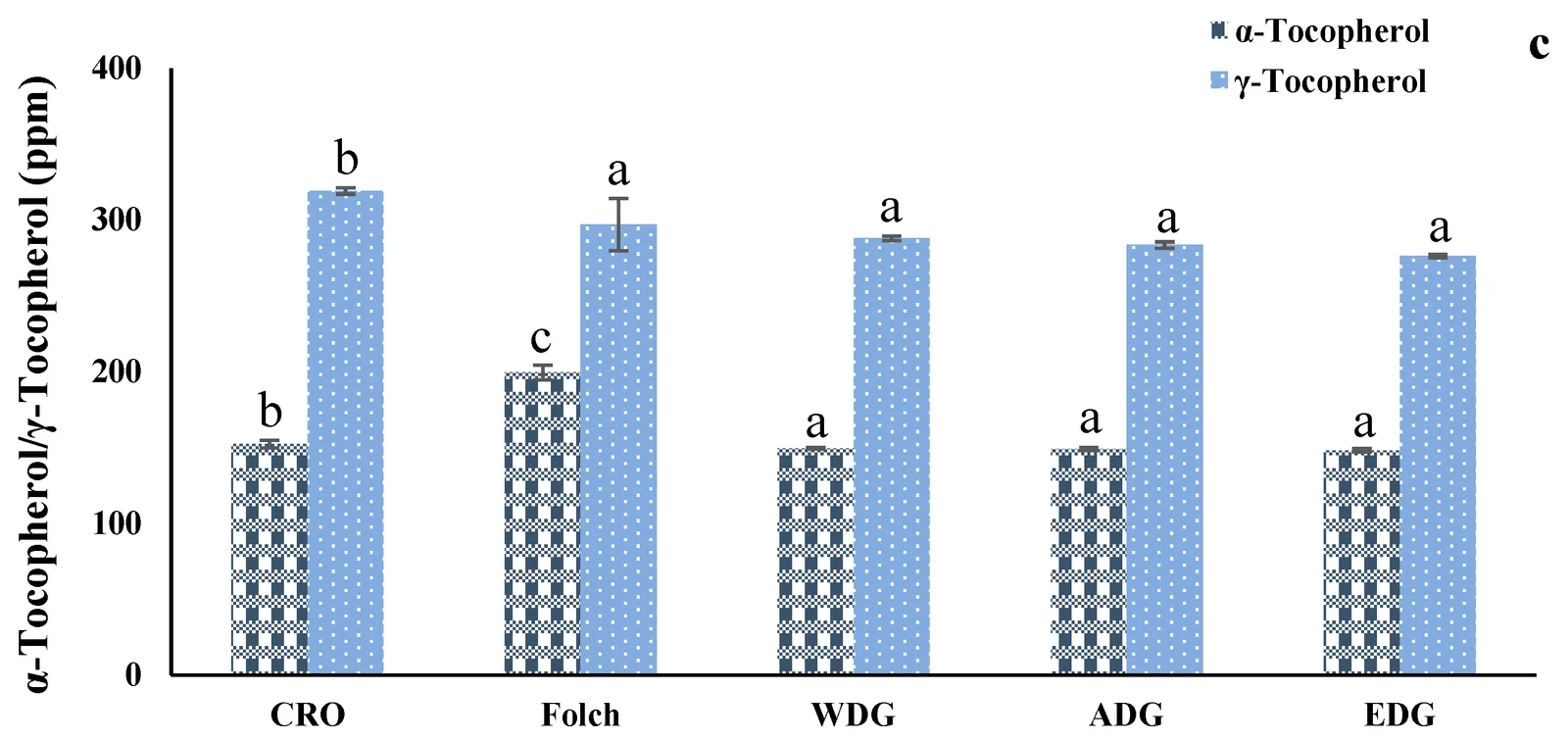
Effects of different degumming processes on the concentrations of phytosterols, carotenoids, and tocopherols in crude and treated rapeseed oils. (**a**) β-Sitosterol and campesterol contents; (**b**) lutein and β-carotene contents; and (**c**) α-tocopherol and γ-tocopherol contents in crude rapeseed oil (CRO) and oils obtained after Folch extraction, water degumming (WDG), citric acid degumming (ADG), and ethanol degumming (EDG). Data are expressed as mean ± standard deviation. Different lowercase letters indicate significant differences among treatments for the same analyte (*p* < 0.05).

The concentrations of lipid-soluble minor components in the recovered oil phases differed among treatments. As shown in Figure 4, crude oil contained the highest levels of lutein, β-carotene, γ-tocopherol, β-sitosterol, and campesterol, and all treatments reduced these components to different extents. Among the degummed oils, WDG showed the highest lutein concentration, whereas Folch-treated oil showed the highest β-carotene concentration among the treated samples. For tocopherols, Folch extraction showed the highest α-tocopherol value, whereas EDG gave the lowest γ-tocopherol among the degummed oils. For phytosterols, WDG showed a slightly higher β-sitosterol concentration than ADG and EDG, while Folch retained campesterol better than the other degumming treatments. These results indicate that degumming altered not only PL recovery but also the retention of nutritionally relevant minor lipids. Because these compounds are highly hydrophobic, their lower concentrations in some treated oils should not be interpreted as direct extraction into the aqueous/alcohol phase. Possible contributions include neutral-oil entrainment in the separated gum phase, association with interfacial aggregates, oxidative degradation or isomerization losses during processing, and differences in analytical recovery. Because these compounds were not quantified in the recovered gum fractions, the present data do not distinguish among these mechanisms.

The decline in carotenoids is consistent with the observed changes in color- and oxidation-related parameters, since these compounds contribute to both antioxidant capacity and visual appearance [1,2,3,4]. Likewise, the tocopherol data show that OIT cannot be explained by tocopherol retention alone, supporting the view that oxidative stability depended on the overall minor-component system rather than on a single antioxidant class [14,15,16,17]. Similar losses of these minor lipid components during oil refining have been reported previously for rapeseed oil and canola oil [2,28,29,30]. The role of vitamin E homologues in oil stability is also well recognized, but their protective effect depends strongly on concentration, matrix composition, and interactions with other components [14,31]. The present results similarly demonstrate process-dependent retention, but extend the analysis by relating the residual oil-phase micronutrients to the amount and composition of the recovered PL-rich fraction. No treatment simultaneously maximized carotenoid, tocopherol, phytosterol, and PL recovery. WDG showed relatively favorable retention of lutein and β-sitosterol, whereas EDG combined moderate oil recovery with a PC- and LPC-enriched PL fraction. These findings demonstrate that residual-oil quality and the compositional value of the recovered fraction represent separate optimization objectives.

### 2.6. Multivariate Analysis Between Crude Rapeseed Oil and Ethanol-Degummed Oil

**Figure 5:**
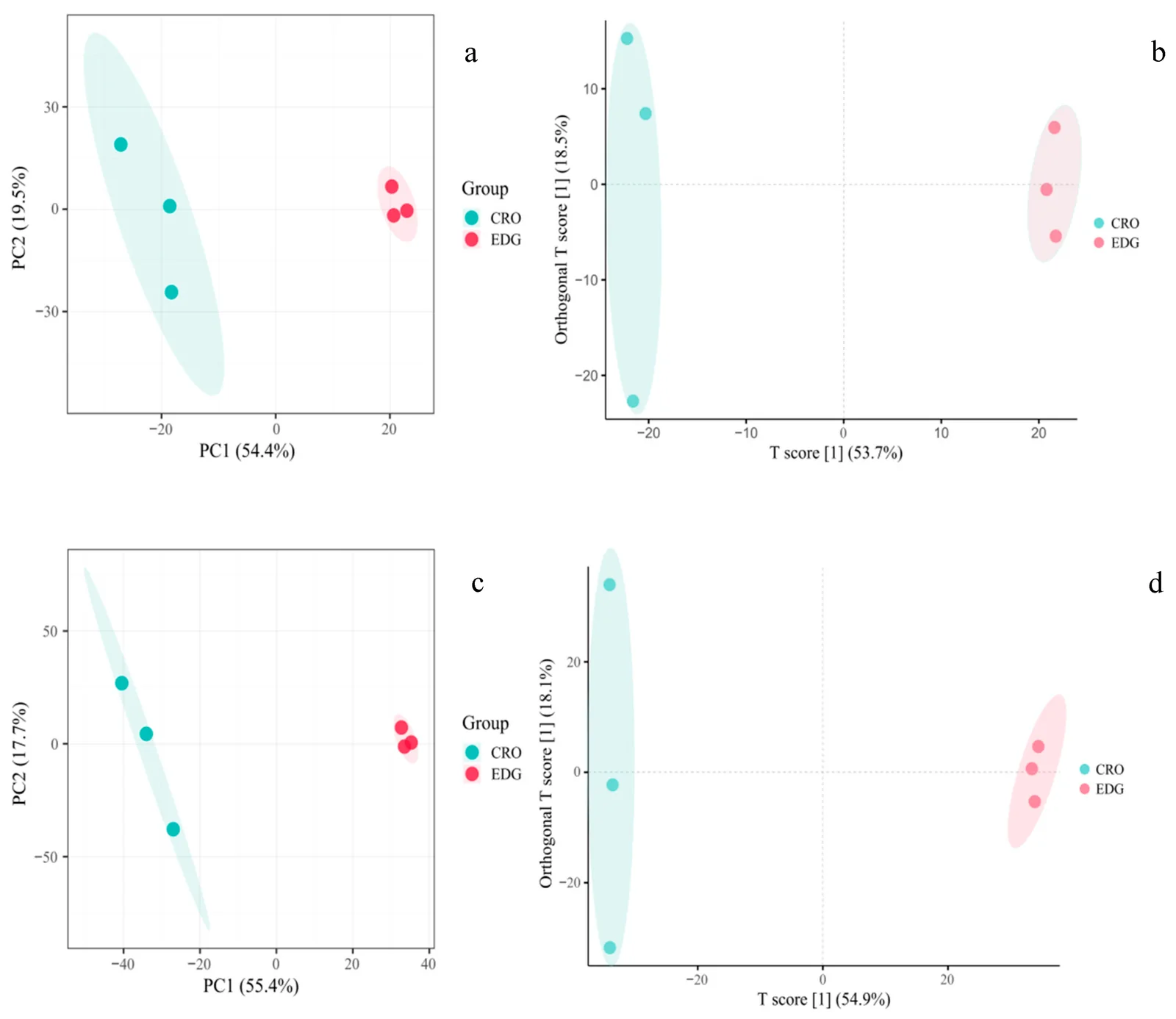
PCA and OPLS-DA score plots showing the compositional discrimination between crude rapeseed oil (CRO) and ethanol-degummed oil (EDG) in positive and negative ion modes. (**a**) PCA score plot in positive ion mode; (**b**) OPLS-DA score plot in positive ion mode; (**c**) PCA score plot in negative ion mode; and (**d**) OPLS-DA score plot in negative ion mode. Clear separation between CRO and EDG indicates that ethanol degumming induced marked changes in the overall metabolite profile.

To further evaluate the compositional effect of ethanol-assisted degumming, multivariate analyses were performed between crude RO and EDG. As shown in Figure 5, both PCA and OPLS-DA score plots showed clear separation between the two groups, indicating that EDG induced a systematic shift in oil composition rather than a small quantitative adjustment. The relatively tight clustering within each group also suggests satisfactory analytical reproducibility.

This separation is consistent with the targeted compositional data discussed above. EDG changed phosphorus content, PL recovery, PL subclass distribution, and the retention of several minor bioactive lipids. The multivariate results therefore support the view that EDG is not simply a mild modification of hydration-based degumming. Instead, it represents a distinct compositional intervention that generates an oil phase and PL-rich fraction with characteristic chemical features. In metabolomics-based food studies, such group separation is generally interpreted as evidence that the treatment affects a coordinated set of metabolites rather than only a few isolated markers [24,32,33].

### 2.7. Metabolite Changes Between Crude Rapeseed Oil and Ethanol-Degummed Oil

**Figure 6:**
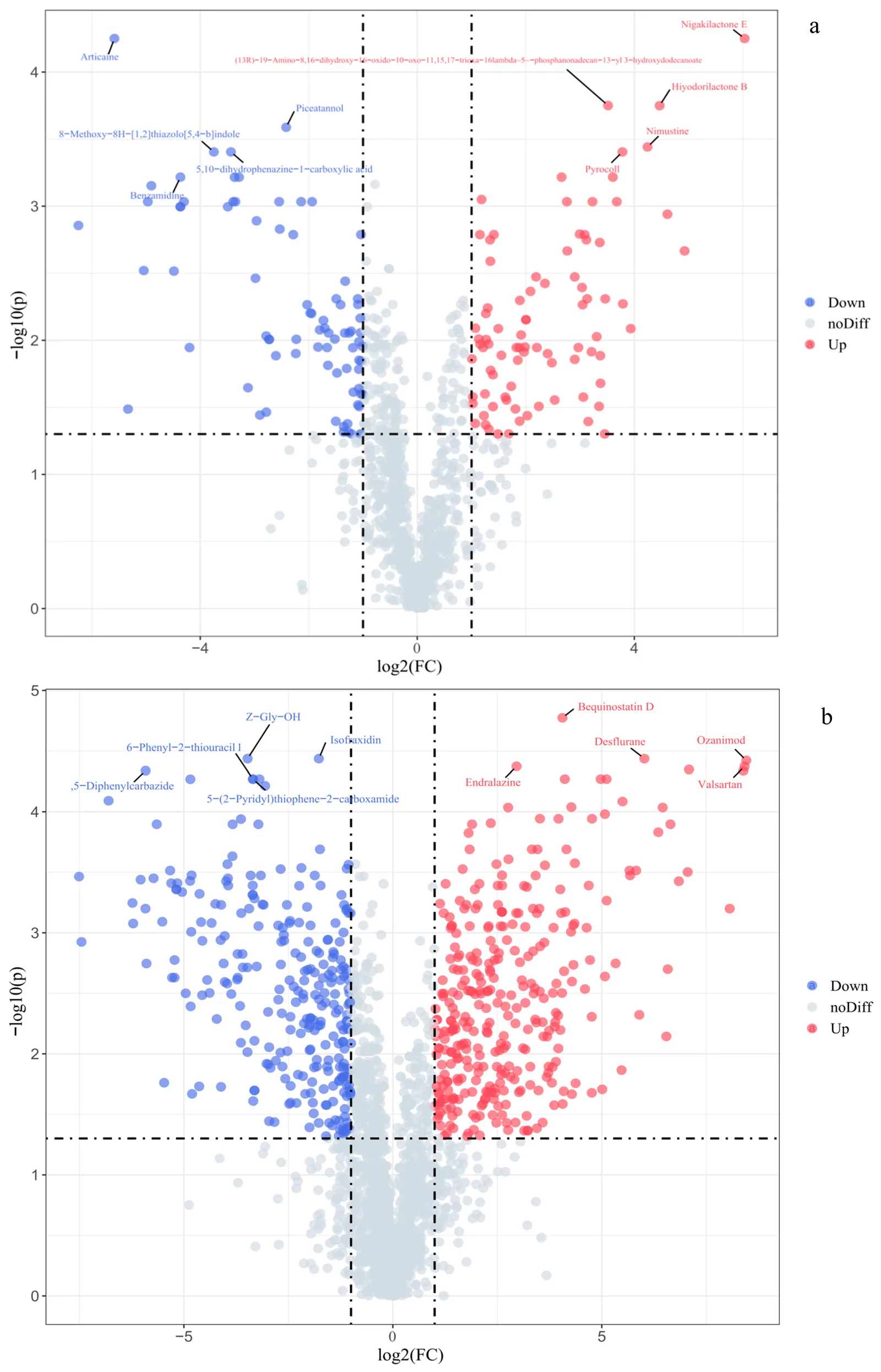
Volcano plots of differential metabolites between crude rapeseed oil (CRO) and ethanol-degummed oil (EDG) in (**a**) positive ion mode and (**b**) negative ion mode. Red and blue points represent significantly upregulated and downregulated metabolites, respectively, while gray points indicate metabolites without significant differences. The vertical dashed lines indicate the fold-change threshold, and the horizontal dashed line indicates the significance threshold.

The untargeted metabolomic results showed that EDG altered the small-molecule profile of rapeseed oil more broadly than could be captured by the targeted indices alone. As shown in Figure 6, volcano plots generated in both negative and positive ion modes revealed large numbers of significantly upregulated and downregulated features in EDG relative to CRO. In negative mode, 79 features were downregulated and 85 were upregulated, whereas in positive mode 271 features were downregulated and 332 were upregulated. The asymmetry between ion modes indicates that ethanol-assisted degumming had a broader effect on the more extensive set of metabolites detected in positive mode, but the key point is that the changes were bidirectional in both modes rather than limited to simple depletion of a few compounds.

This pattern suggests that ethanol-assisted degumming did not merely strip polar compounds from the oil phase. Instead, it redistributed a wider pool of metabolites, likely including amphiphilic lipids, oxygenated minor components, and other moderately polar constituents whose partitioning is sensitive to solvent polarity. In practical terms, this means that EDG changed the chemical environment of the oil more broadly than would be inferred from phosphorus removal or PL yield alone. Such interpretation is consistent with current metabolomics practice, where volcano plots, together with PCA and OPLS-DA, are used to characterize treatment-associated remodeling at the systems level rather than to emphasize only single compounds [24,32,33].

### 2.8. Lipidomic Remodeling of Phospholipid Subclasses After Ethanol Degumming

**Figure 7:**
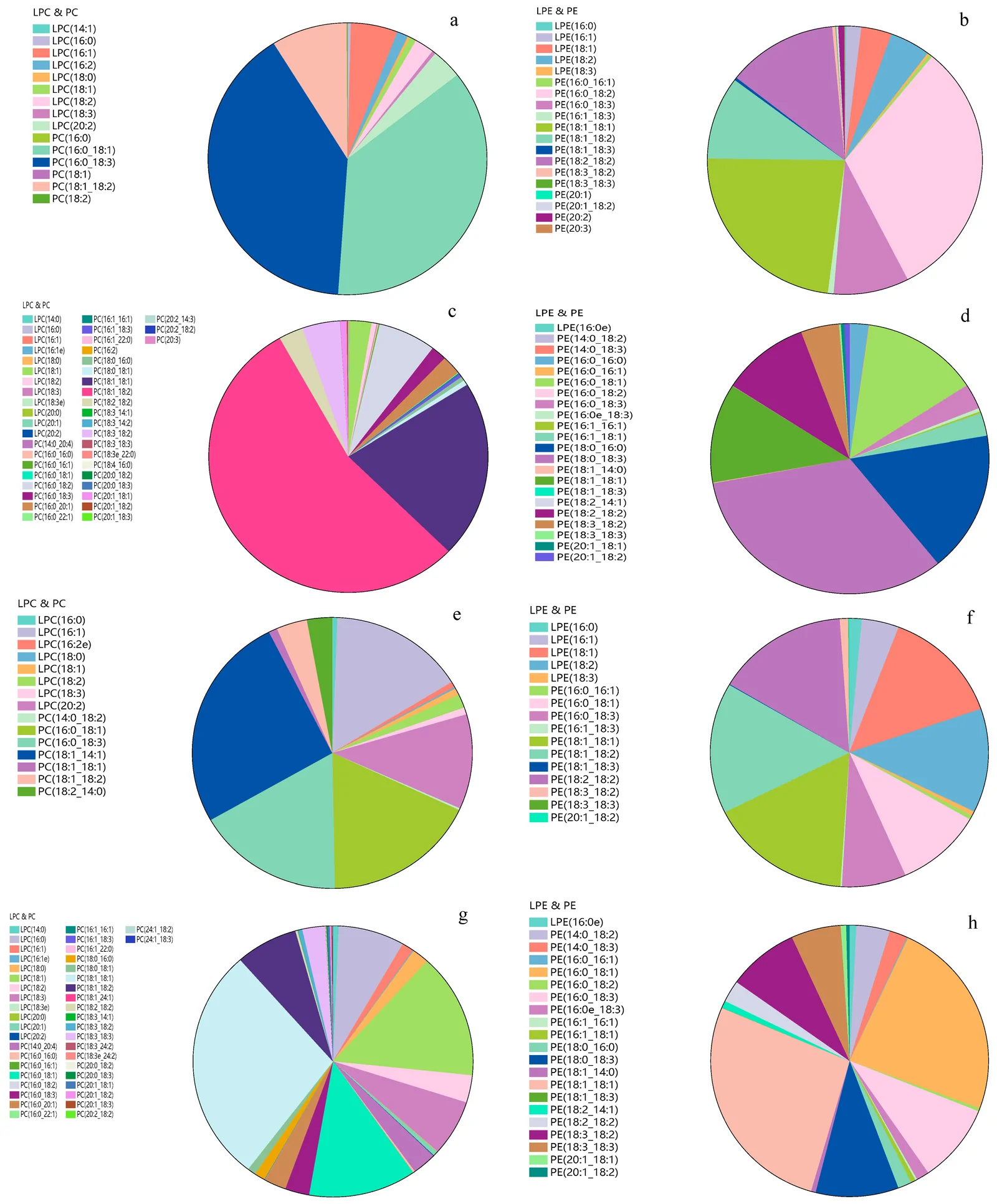
Fatty acid profiles of LPC & PC, LPE & PE in CRO (**a**–**d**, respectively) and EDG (**e**–**h**, respectively) acquired in positive and negative ion modes. Pie charts show the proportional distribution of individual molecular species within each phospholipid subclass.

The lipidomic data provided additional resolution at the level of PL molecular species. As shown in Figure 7, EDG altered the relative abundance of individual molecular species within the major PL subclasses compared with CRO. This result is important because changes in total PL class abundance do not fully describe PL functionality. Molecular species differ in acyl-chain composition, unsaturation level, polarity, and potential interfacial behavior, and these features are likely to affect both physicochemical performance and nutritional relevance.

The species-level redistribution observed here indicates that EDG was selective not only at the subclass level but also at the molecular-species level. This agrees with the PL class data in Figure 2a, where EDG favored PC- and LPC-containing fractions, but the lipidomic results show that the effect went further, reshaping the internal composition of these subclasses. This point is particularly important because food lipidomics studies increasingly show that processing effects may be modest at the class level but pronounced at the species level, especially for PLs and other minor lipid groups [25,34,35]. From an application standpoint, the present data therefore suggest that EDG may be useful not only for PL recovery but also for obtaining PL-rich fractions with more specific molecular features.

### 2.9. Correlation and Hierarchical Clustering Analysis

**Figure 8:**
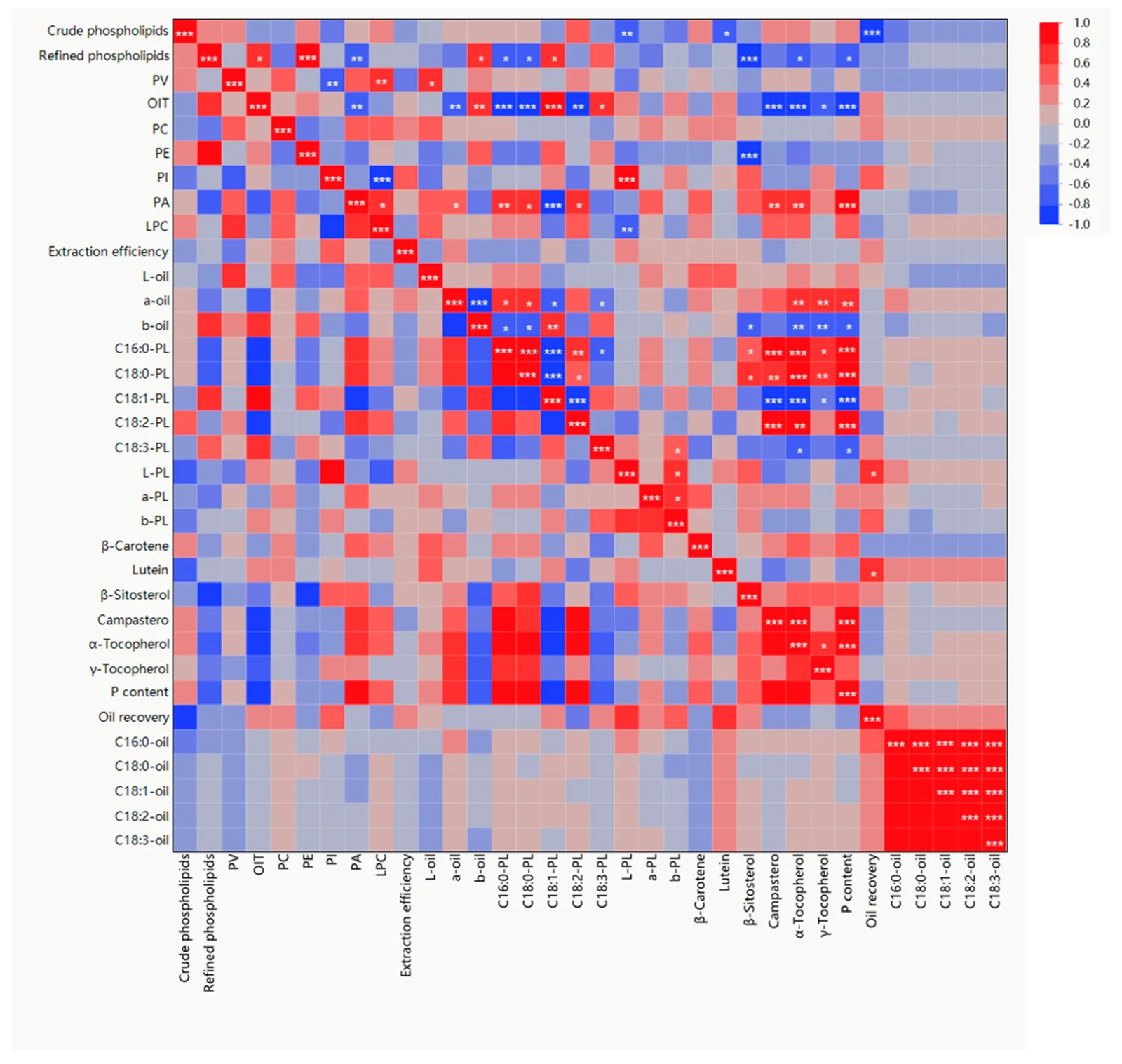
Heatmap showing the Pearson correlation among phospholipid fractions, phospholipid subclasses, physicochemical properties, bioactive lipid components, and fatty acid composition of rapeseed oil samples after different degumming methods. *, **, and *** indicate significance at the 95%, 99%, and 99.9% confidence levels, respectively.

Pearson correlation analysis was performed using the individual process replicates rather than treatment means. The heatmap revealed close relationships among PL fractions, PL classes, oil quality parameters, carotenoids, phytosterols, tocopherols, phosphorus content, and oil recovery. As shown in Figure 8, these associations indicate that PL removal, retention of bioactive lipids, and oil quality were tightly connected outcomes rather than independent responses. This is consistent with the current understanding of vegetable oil refining and oxidation, where PLs, antioxidants, pigments, trace metals, and oxidation products all interact within the same matrix [14,15,16,17].

**Figure 9:**
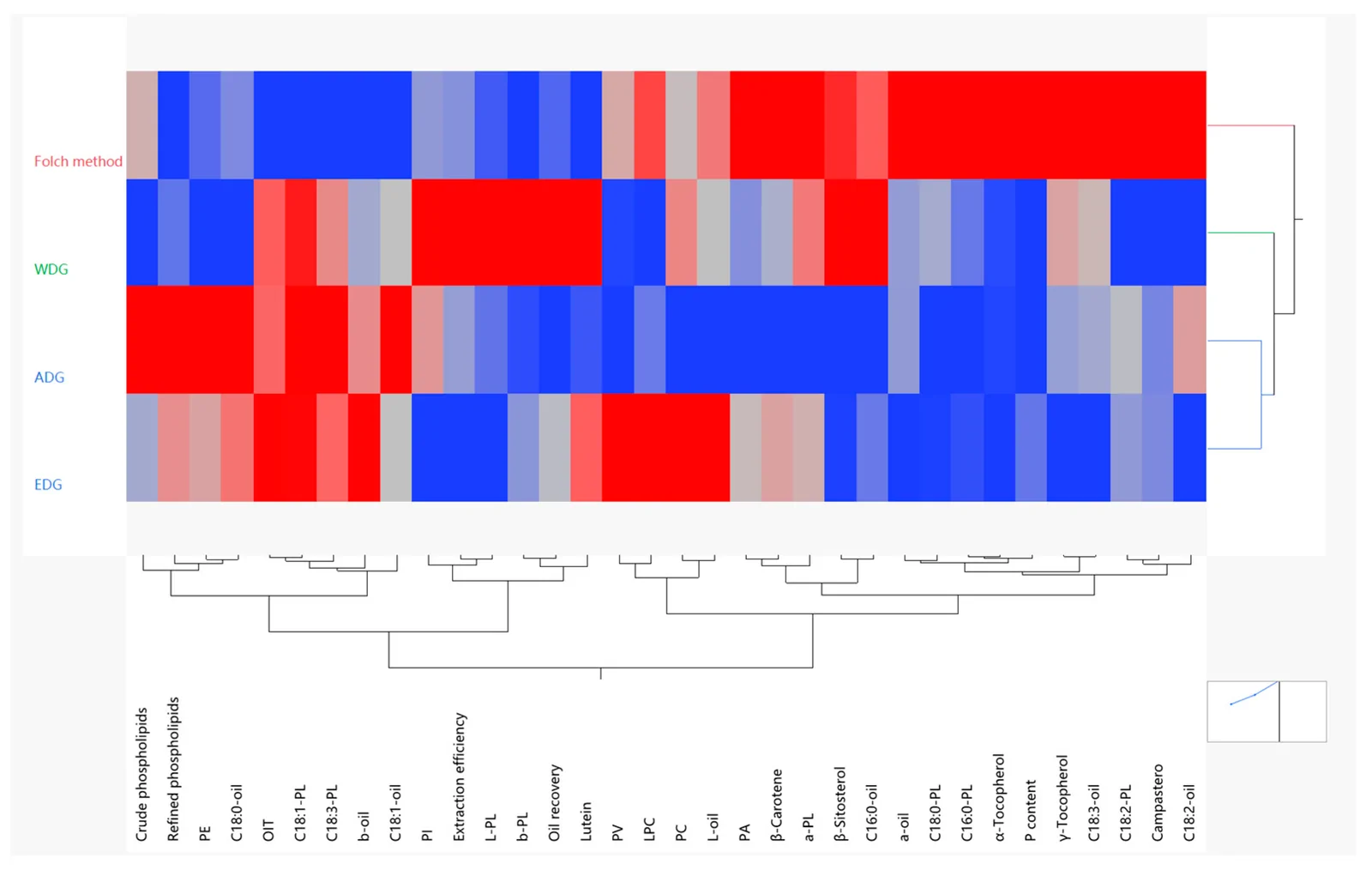
Hierarchical clustering heatmap showing the compositional differences among Folch extraction, water degumming (WDG), citric acid degumming (ADG), and ethanol degumming (EDG) based on phospholipid fractions, bioactive lipid components, physicochemical properties, and fatty acid composition. Red and blue colors indicate relatively high and low normalized levels, respectively.

The hierarchical clustering heatmap further showed that the different treatments generated clearly distinct compositional fingerprints. As shown in Figure 9, ADG and EDG diverged more strongly from Folch and WDG, indicating that acid- and ethanol-assisted systems imposed greater compositional changes on the lipid matrix. This integrated analysis reinforces the conclusion that each degumming process should be regarded as a distinct compositional intervention rather than as a simple variation of the same refining step.

### 2.10. Comparison with Previous Studies and Overall Assessment

Previous rapeseed oil studies have established the effects of water, acid, enzymatic, and electrolyte-assisted degumming on residual phosphorus, oil yield, oxidative stability, bioactive component retention, and PL composition [18,23,24,28]. The present study does not challenge these established process principles. Instead, it extends them through an integrated comparison of the treated oil and the recovered PL-rich fraction, with particular emphasis on ethanol-assisted degumming. By combining oil recovery, PL yield and purity, PL subclass and FA distributions, morphology, micronutrient retention, and molecular-level profiling, the results distinguish three process objectives that would not be apparent from residual phosphorus alone.

WDG was preferable when oil recovery, process simplicity, and PL purity were prioritized. ADG achieved the highest PL recovery but involved greater oil loss. EDG did not maximize either dephosphorization or individual micronutrient retention; its distinguishing feature was the combination of moderate PL recovery, relatively high oil recovery, enrichment of PC and LPC, and a characteristic metabolomic and lipidomic profile. Therefore, the principal contribution of this study is not the confirmation that degumming changes rapeseed oil composition, but the demonstration that different processes direct distinct balances between refining efficiency, preservation of the oil phase, and compositional characteristics of the recovered PL fraction.

However, the present comparison was conducted at laboratory scale using the same crude-oil batch and controlled processing conditions. The results should therefore be interpreted as comparative compositional data rather than as direct validation of industrial process performance. Pilot- and/or industrial-scale studies using comparable continuous processing equipment will be required to assess scale-up, solvent recovery, energy demand, process safety, and economic feasibility, particularly for EDG.

## 3. Materials and Methods

### 3.1. Materials

Crude RO was obtained from Qinghai Tongda Oil Processing Co., Ltd. (Xining, China). The oil was produced from the Qingza 5 rapeseed varieties grown in Huzhu Tu Autonomous County, Qinghai Province, China, and was obtained by mechanical pressing after roasting. The rapeseed seeds were harvested in September 2025. The oil extraction process was conducted by the aforementioned company. The oil was produced in October 2025 and shipped to the laboratory on the second day of the extraction. After arrival, the crude RO was stored in sealed amber glass bottles at −60 °C before use. The same batch of crude oil was used for all experiments.

A soybean PL mixture, β-sitosterol, brassicasterol, and campesterol were purchased from Sigma-Aldrich (Shanghai, China). Lutein, β-carotene, α-tocopherol, γ-tocopherol, δ-tocopherol, oleic acid, glyceryl monooleate, glyceryl dioleate, and triolein were obtained from Macklin (Shanghai, China). The solvents used in this study were high-performance liquid chromatography (HPLC)-grade and purchased from local chemical vendors.

### 3.2. Degumming Procedures and Laboratory Reference Extraction

WDG was performed with minor modifications according to the previous methods [4,18,20]. Briefly, crude RO (200 g) was heated to 80 °C, followed by the addition of deionized water (6 mL) under high-shear homogenization at 10,000 rpm for 1 min using a high-shear homogenizer. The mixture was then stirred at 500 rpm for 20 min to promote hydration and aggregation of hydratable PLs. After treatment, the gum phase was separated from the degummed oil by centrifugation at 10,000 rpm for 10 min. The separated gum fraction was collected for subsequent PL recovery and analysis. The water dosage of 3 mL/100 g oil was selected based on reported water-degumming procedures for rapeseed oil [18] and preliminary bench-scale trials. This dosage was sufficient to hydrate the hydratable PL fraction while limiting excessive emulsion formation and neutral-oil entrainment. The dosage was used as a fixed comparative condition and was not subjected to formal optimization in the present study.

ADG was performed with slight modifications based on reported acid-degumming principles and procedures [4,5,20]. Crude RO (200 g) was first heated to 80 °C, and 6 mL of 45% (*w*/*w*) aqueous citric acid solution was then added under high-shear mixing at 10,000 rpm for 1 min. The mixture was stirred at 80 °C for 30 min for acid treatment. After acidification, 1 mL of deionized water was added and homogenized at 10,000 rpm for 1 min, followed by further stirring at 80 °C for 20 min. The gum phase and degummed oil were then separated by centrifugation at 10,000 rpm for 10 min. For recovery of the crude PL fraction, the centrifuge was precooled to below −5 °C before the final centrifugation step. After centrifugation, the gum layer was carefully collected and concentrated by rotary evaporation to remove water. The residue was then extracted several times with 50 mL portions of chloroform, and the combined chloroform extracts were evaporated to dryness to obtain the crude PL fraction. The citric acid concentration and dosage were selected based on reported acid-degumming principles and procedures [4,5,8,20] and preliminary separation trials. These conditions were used to disrupt metal–PL associations and convert non-hydratable phospholipids into more readily hydratable and separable forms [4,5,8,20]. No formal response-surface optimization was performed; therefore, the selected conditions should be regarded as fixed comparative parameters rather than universal optima. Because the system consisted predominantly of a nonaqueous oil phase with a limited dispersed aqueous phase, conventional bulk pH was not used as a process-control parameter. Citric acid concentration and dosage were controlled directly.

For EDG, crude RO (200 g) was mixed with 40 mL of 70% aqueous ethanol (*v*/*v*) under high-shear mixing at 10,000 rpm for 1 min. The solvent-to-oil ratio was therefore 20 mL/100 g oil. The 70% ethanol solution was prepared from absolute ethanol and deionized water at 7:3 (*v*/*v*) and precooled to −20 °C before addition. The stated temperature refers to the ethanol solution before mixing rather than the equilibrium temperature of the complete oil–ethanol system. After solvent addition, the temperature of the mixture was around 15 °C.

Precooling was used to favor aggregation of the PL-rich phase and to limit co-solubilization of neutral oil during subsequent separation. The mixture was stirred at 2000 rpm for 5 min and centrifuged at 10,000 rpm for 10 min using a refrigerated centrifuge set at −10 °C. Refrigerated centrifugation was used to maintain the aggregated PL-rich phase and reduce redissolution during phase separation. Cooling may also increase the viscosity of the oil-rich phase and slow mass transfer; therefore, the selected temperature and centrifugal conditions represent a compromise between PL aggregation and efficient phase separation.

The effects of solvent temperature and centrifugation temperature were not independently optimized in this study. Accordingly, differences in recovered PL subclasses are interpreted as outcomes of the complete EDG process rather than as effects of cooling alone.

Folch extraction was selected as a commonly used laboratory solvent-extraction reference method for comparison with the three degumming processes. It was included to provide a reference for the compositional data of the recovered lipid fractions rather than to represent an industrial degumming alternative. Other laboratory extraction procedures were not evaluated, as comparison among lipid-extraction methods was outside the scope of the present study. Ten grams of crude RO was mixed with 75 mL of a mixed solvent of chloroform/methanol (2/1, *v*/*v*); then, 25 mL of water was added for phase separation. The PL-containing methanol/water phase layer was then collected and the solvent was evaporated by a rotary evaporator. The Folch procedure was not treated as an industrial degumming method because chloroform–methanol extraction is neither food-compatible nor directly comparable with aqueous or acid-assisted oil degumming. It was included only to provide a laboratory compositional benchmark.

### 3.3. Determination of Oil Quality, Phospholipid Characteristics, and Minor Lipid Components

Total phosphorus was determined according to the American Oil Chemists’ Society (AOCS) Official Method Ca 12-55 [36] on a Metash X8s spectrophotometer (Shanghai Metash Instruments, Shanghai, China).

FA compositions of crude RO and degummed oil were determined with slight modifications based on the procedure described by Wu et al. [2] using an Agilent 6890 gas chromatograph coupled to an Agilent 5973 mass selective detector (Agilent, Santa Clara, CA, USA). Separation of fatty acid methyl esters (FAMEs) was achieved on a VF-17ms column (30 m × 0.25 mm × 0.25 μm; Agilent, Santa Clara, CA, USA) using helium as the carrier gas. FAs were identified and quantified using an external 37-component FAME standard mixture (Supelco Inc., Bellefonte, PA, USA).

MAGs, DAGs, PL subclasses, tocopherols, β-sitosterol, campesterol, lutein, and β-carotene were determined using a comprehensive HPLC method developed with reference to previous reports [25,28,34]. Analyses were performed on a Waters e2695 HPLC system (Waters Corporation, Milford, MA, USA) equipped with a photodiode array detector (PDA) and an evaporative light-scattering detector (ELSD, Alltech Leader Technology Co., Ltd., Chengdu, China). Separation was carried out on a Morphling^®^ WD-SiO_2_ column (HeXi Biotechnology Co., Ltd., Nanjing, China). The mobile phase consisted of solvent A, n-hexane containing 2% acetic acid (*v*/*v*); solvent B, ethyl acetate; and solvent C, isopropanol. The gradient program was as follows: 0–45 min, from 100% A to 85% A/15% B; 45–65 min, linear increase to 100% B; 65–66 min, switched to 100% C; and 66–70 min, held at 100% C. The flow rate was 0.8 mL/min, the injection volume was 10 μL, and the column temperature was maintained at 35 °C. PDA spectra were recorded over 190–700 nm, with 450 nm used for carotenoids and 294 nm for tocopherols. Non-UV-absorbing compounds, including FFAs and acylglycerols, were detected by ELSD at 40 °C with a nitrogen pressure of 3.5 bar.

For sample preparation, 100 mg of sample was accurately weighed, dissolved in chloroform/methanol (1:1, *v*/*v*), and diluted to a final concentration of 10 mg/mL. The solution was centrifuged at 10,000 rpm for 5 min and filtered through a 0.22 μm hydrophobic polytetrafluoroethylene (PTFE) syringe filter before injection. Quantification was performed using external standards, and analytes were identified by comparison of retention times with those of authentic standards.

### 3.4. Analysis of Physical Properties of Crude Oil and Phospholipids

The microstructural morphology of the recovered PL fractions was characterized at room temperature using a PLM (MSD-S820, Murzider Technology Co., Ltd., Dongguan, China) and an SEM (JSM-6360LV, JEOL, Tokyo, Japan). For PLM, a small amount of sample was spread evenly on a glass slide, covered with a coverslip, and observed under polarized light at appropriate magnifications. For SEM analysis, samples were mounted on conductive adhesive tape, coated with platinum, and examined at an accelerating voltage of 15 kV using both secondary electron imaging (SEI) and backscattered electron imaging (BEI) modes.

The apparent color of the oil samples and recovered PL-rich fractions was measured using a colorimeter (3NH Technology Co., Ltd., Shenzhen, China). The oil fractions were measured in the state obtained after the corresponding separation process, without additional clarification before color analysis. Some samples, particularly the Folch- and ADG-treated oils, retained visible turbidity and suspended material.

Samples were transferred into 10 mm quartz cuvettes and measured under a D65 standard illuminant with a 10° observer angle. Color was expressed in the CIE La*b* color space, where L* represents apparent lightness, a* represents the red–green coordinate, and b* represents the yellow–blue coordinate. Because residual turbidity influences light scattering, the reported values represent the apparent visual characteristics of the processed samples rather than pigment-related color alone.

### 3.5. Evaluation of Oxidative Stability

Primary oxidation products were determined as PV according to the Association of Official Analytical Chemists (AOAC) Official Method 965.33 [37].

Thermal oxidative stability was further evaluated by differential scanning calorimetry (DSC; Setaram Setline, KEP Technologies, Plan-les-Ouates, Switzerland) with slight modification of a previously reported method [38]. Approximately 10 mg of sample was sealed in an aluminum pan with a punctured lid. The sample was first heated from 25 to 140 °C at 30 °C/min under nitrogen at 50 mL/min and held isothermally at 140 °C for 6 min. The purge gas was then switched to oxygen at 50 mL/min, and the sample was maintained at 140 °C for 120 min. After oxidation, the sample was cooled from 140 to 25 °C at 50 °C/min and held at 25 °C for 1 min. The oxidation induction time (OIT) was determined from the onset of the exothermic oxidation peak after switching from nitrogen to oxygen.

### 3.6. Metabolomic and Lipidomic Analyses

Untargeted metabolomic analysis was performed using liquid chromatography–mass spectrometry (LC-MS, UltiMate 3000, Thermo Scientific, Waltham, MA, USA; Orbitrap Exploris 480, Thermo Scientific, Waltham, MA, USA). Briefly, 200 μL of each sample was mixed with 10 μL of internal standard solution (100 ppm L-2-chlorophenylalanine) and 800 μL of methanol/acetonitrile (1:1, *v*/*v*). The mixture was vortexed for 1 min, sonicated for 30 min, and centrifuged at 12,000 rpm and 4 °C for 5 min. The supernatant was concentrated under vacuum for 4 h, reconstituted in 200 μL of 50% aqueous methanol, vortexed, sonicated, filtered, and subjected to LC-MS analysis.

Chromatographic separation was performed on an HSS T3 column (Waters, Milford, MA, USA) at a flow rate of 0.4 mL/min, an injection volume of 1 μL, and a column temperature of 50 °C. For positive ion mode, water containing 0.1% formic acid and acetonitrile containing 0.1% formic acid were used as mobile phases A and B, respectively. For negative ion mode, water and acetonitrile were used as mobile phases A and B, respectively. MS analysis was conducted using heated electrospray ionization (HESI) in both positive and negative modes, with a scan range of *m*/*z* 67–1000. Raw data were processed using Compound Discoverer software (version 3.3; Thermo Scientific, Waltham, MA, USA), and metabolite annotation was performed by matching accurate mass and MS/MS spectra against mzCloud, mzVault, and ChemSpider databases. Quality control (QC) samples were prepared by pooling equal aliquots of all samples, and metabolites with a QC coefficient of variation (CV) < 30% were retained for subsequent analysis.

Untargeted lipidomic analysis was performed using ultra-high-performance liquid chromatography (UHPLC) coupled with Orbitrap mass spectrometry (UHPLC–Orbitrap MS; UHPLC Nexera LC-30A, Shimadzu, Kyoto, Japan; Q Exactive HF-X, Thermo Scientific, Waltham, MA, USA). QC samples were prepared by pooling equal aliquots of all samples and were inserted throughout the analytical sequence to monitor system stability and data reproducibility.

An aliquot of 100 μL of each sample was mixed with 240 μL of precooled methanol (−20 °C), vortexed, and extracted with 800 μL of methyl tert-butyl ether (MTBE). The mixture was vortexed, sonicated in a cold-water bath for 20 min, kept at room temperature for 30 min, and centrifuged at 14,000× *g* and 10 °C for 15 min using a refrigerated centrifuge (5430R, Eppendorf, Hamburg, Germany). The upper organic phase was collected and dried under nitrogen. Before LC-MS/MS analysis, the dried residue was reconstituted in 200 μL of 90% isopropanol/acetonitrile, vortexed, and centrifuged again at 14,000× *g* and 10 °C for 15 min. The supernatant was used for lipidomic analysis.

Chromatographic separation was carried out on an ACQUITY UPLC CSH C18 column (1.7 μm, 2.1 mm × 100 mm; Waters, Milford, MA, USA). Samples were maintained at 8 °C in the autosampler. The injection volume was 2 μL, the column temperature was 50 °C, and the flow rate was 0.3 mL/min. Mobile phase A consisted of acetonitrile/water (3:2, *v*/*v*), and mobile phase B consisted of isopropanol/acetonitrile (9:1, *v*/*v*). The gradient elution program was as follows: 0–3.5 min, 40–43% B; 3.5–4.0 min, 43–50% B; 4.0–7.0 min, 50–60% B; 7.0–13.0 min, 60–75% B; 13.0–17.0 min, 75–99% B; 17.0–19.0 min, 99% B; 19.0–19.5 min, 99–40% B; and 19.5–24.0 min, 40% B.

MS detection was performed using ESI in both positive and negative ion modes. The MS parameters were as follows: sheath gas flow rate, 30 arbitrary units; auxiliary gas flow rate, 10 arbitrary units; spray voltage, 2.5 kV in both positive and negative ion modes; S-lens RF level, 50; capillary temperature, 325 °C; auxiliary gas temperature, 300 °C; normalized collision energy, 30; isolation window, 1.5 *m*/*z*; TopN, 10; and scan range, *m*/*z* 200–1800.

Data were processed using LipidSearch software (version 4.1; Thermo Scientific, Waltham, MA, USA) and R software (version 4.3.1; R Foundation for Statistical Computing, Vienna, Austria).

### 3.7. Statistical Analysis

The study used a single-factor experimental design, with treatment method as the independent factor. Each degumming treatment was independently conducted three times using separate oil portions, resulting in three independent process replicates per treatment.

Results are expressed as mean ± standard deviation (SD) of the three independent process replicates. Data were analyzed using one-way analysis of variance (ANOVA), followed by Tukey’s honest significant difference test (HSD) for pairwise comparisons. Tukey’s procedure was used to control the family-wise error rate; therefore, no additional multiple-comparison correction was applied to the targeted physicochemical and compositional variables. Statistical significance was defined as *p* < 0.05.

Potential outliers were evaluated by reviewing the original analytical records, calibration results, sample-preparation records, and instrumental quality-control information. No observations were removed solely because of their numerical distance from the group mean. No values in the targeted analyses were excluded as statistical outliers.

Statistical analysis was performed using JMP^®^ software (version 17.1, SAS Institute Inc., Cary, NC, USA) and SPSS Statistics (version 25, IBM Corp., Armonk, NY, USA).

## 4. Conclusions

The different degumming processes produced clearly different compositional and quality outcomes in crude RO. WDG, ADG, and EDG produced distinct outcomes in terms of phosphorus removal, oil recovery, and PL recovery. WDG provided the highest oil recovery and PL purity, whereas ADG gave the highest PL recovery yield. EDG showed an intermediate balance between oil recovery and PL recovery and yielded a PL-rich fraction with relatively high PC and LPC contents. The bulk fatty acid composition remained largely unchanged, while PL subclass distribution, minor-lipid concentrations, morphology, and molecular profiles were treatment-dependent.

These results demonstrate that degumming-method selection should consider both refining efficiency and the composition of the recovered PL-rich fraction rather than residual phosphorus alone. The metabolomic and lipidomic analyses revealed broad compositional differences between crude and ethanol-degummed oils; however, further mass-balance, storage-stability, interfacial-property, and functional studies are required to determine the practical significance of these differences.

## Figures and Tables

**Table 1:** Physicochemical characteristics and lipid composition of rapeseed oil after different degumming processes.

Extraction Methods		Crude Rapeseed Oil	Folch	WDG	ADG	EDG
Fatty acid (%)	C16:0	3.98 ± 0.30 ^a^	4.02 ± 0.16 ^a^	4.04 ± 0.31 ^a^	3.93 ± 0.28 ^a^	3.97 ± 0.27 ^a^
	C18:0	4.5 ± 0.32 ^a^	4.53 ± 0.34 ^a^	4.51 ± 0.27 ^a^	4.58 ± 0.32 ^a^	4.56 ± 0.36 ^a^
	C18:1	57.48 ± 1.03 ^a^	57.47 ± 1.09 ^a^	57.48 ± 1.01 ^a^	57.49 ± 1.06 ^a^	57.48 ± 1.04 ^a^
	C18:2	24.39 ± 1.23 ^a^	24.40 ± 1.13 ^a^	24.38 ± 1.20 ^a^	24.39 ± 1.25 ^a^	24.38 ± 1.27 ^a^
	C18:3	6.59 ± 1.10 ^a^	6.65 ± 1.07 ^a^	6.61 ± 1.09 ^a^	6.60 ± 1.13 ^a^	6.57 ± 1.07 ^a^
Monoacylglycerols (ppm)		537.17 ± 3.45 ^d^	310.57 ± 1.98 ^b^	306.73 ± 0.60 ^b^	291.47 ± 4.24 ^a^	322.30 ± 5.57 ^c^
Diacylglycerols (ppm)		4147.57 ± 124.58 ^c^	3295.20 ± 123.25 ^a^	3746.57 ± 85.48 ^b^	3729.87 ± 30.30 ^b^	3602.70 ± 92.21 ^b^
Phosphorus content (mg/g)		0.249 ± 0.017 ^d^	0.185± 0.004 ^c^	ND ^a^	ND ^a^	0.022 ± 0.001 ^b^
PV (mEq/kg)		0.37 ± 0.20 ^a^	2.79 ± 0.12 ^bc^	1.79 ± 0.36 ^ab^	1.74 ± 0.44 ^ab^	4.02 ± 1.37 ^c^
OIT (min)		181.13 ± 1.47 ^d^	57.60 ± 1.57 ^a^	101.37 ± 1.70 ^b^	100.53 ± 0.65 ^b^	108.90 ± 0.79 ^c^
Colorimetry (Oil)	L	27.13 ± 0.19 ^ab^	25.80 ± 2.04 ^ab^	22.85 ± 1.72 ^ab^	12.41 ± 10.64 ^a^	30.20 ± 7.59 ^b^
	a*	1.24 ± 0.12 ^ab^	2.95 ± 2.04 ^b^	0.70 ± 0.035 ^ab^	0.69± 0.34 ^ab^	−0.15 ± 0.79 ^a^
	b*	2.11 ± 0.16 ^b^	−3.71 ± 3.65 ^a^	0.26 ± 0.544 ^ab^	2.58 ± 2.57 ^b^	5.76 ± 1.59 ^b^

ND, not detected; Each test was carried out in triplicate, and the data were expressed as mean ± standard deviation. Different letters indicate significant statistical difference at *p* < 0.05 in Tukey’s honest significant difference (HSD) test. Color parameters represent the apparent color of the oil fractions as obtained after processing. Residual turbidity, particularly in the Folch and ADG samples, contributed to the observed variability.

## Data Availability

The original contributions presented in this study are included in the article. Further inquiries can be directed to the corresponding author.

## Abbreviations

The following abbreviations are used in this manuscript:

ADG	acid-assisted degumming
ANOVA	analysis of variance
AOAC	Association of Official Agricultural Chemists
AOCS	American Oil Chemists’ Society
ATR	attenuated total reflectance
BEI	backscattered electron imaging
CV	coefficient of variation
DAG	diacylglycerol
DSC	differential scanning calorimetry
EDG	ethanol degumming
ELSD	evaporative light-scattering detector
FAs	fatty acids
FFAs	free fatty acids
FAME	fatty acid methyl esters
HESI	heated electrospray ionization
HPLC	high-performance liquid chromatography
HSD	honest significant difference
LC-MS	liquid chromatography–mass spectrometry
LPC	lysophosphatidylcholine
MAG	monoacylglycerol
MS	mass spectrometry
MTBE	methyl tert-butyl ether
OIT	oxidation induction time
OPLS-DA	orthogonal partial least squares-discriminant analysis
PA	phosphatidic acid
PC	phosphatidylcholine
PCA	principal component analysis
PDA	photodiode array detector
PE	phosphatidylethanolamine
PI	phosphatidylinositol
PL	phospholipid
PLs	phospholipids
PTFE	polytetrafluoroethylene
PV	peroxide value
QC	quality control
RO	rapeseed oil
SD	standard deviation
SEI	secondary electron imaging
SEM	scanning electron microscopy
TAG	triacylglycerol
UHPLC	ultra-high-performance liquid chromatography
VIP	variable importance in projection
WDG	water degumming
